# Development and psychometric evaluation of College Students’ Employment Stress Scale based on five-dimensional structure: a case study of Zhejiang University of Science and Technology

**DOI:** 10.3389/fpsyg.2026.1763829

**Published:** 2026-06-01

**Authors:** Minhui Hu

**Affiliations:** Institutional Resources of Civil Engineering and Architectural, Zhejiang University of Science and Technology, Hangzhou, Zhejiang, China

**Keywords:** college students, employment pressure, mental health, reliability and validity, scale development

## Abstract

**Introduction:**

This study aims to develop a standardized scale for measuring contemporary college students’ employment stress and to evaluate its psychometric properties.

**Methods:**

Through literature review, open-ended questionnaires (*n* = 30), and in-depth interviews (*n* = 15), an initial item pool of 31 items was constructed. Using stratified sampling, undergraduate students from Zhejiang University of Science and Technology (ZUST) were recruited for preliminary testing (113 valid samples) and formal testing (234 valid samples). SPSS 28.0 was employed for item analysis and exploratory factor analysis, while AMOS 21.0 was used for confirmatory factor analysis.

**Results:**

The final scale comprised 23 items across five dimensions: Personal Qualities, Family Support, Job Market & Major Utility, Institutional Resources, and Regional Factors, together explaining 64.46% of the total variance. Confirmatory factor analysis indicated marginal model fit (*χ*^2^/df = 3.942, RMSEA = 0.113, CFI = 0.79), suggesting that the hypothesized five-factor structure requires further refinement. Preliminary evidence of criterion-related validity was obtained through a weak but statistically significant correlation with the Self-Rating Anxiety Scale (*r* = 0.151, *p* < 0.05).

**Discussion:**

The scale demonstrates acceptable internal consistency at the full-scale level; however, its structural validity remains marginal and the Institutional Resources subscale is psychometrically inadequate in its current form. The scale should be regarded as a preliminary research instrument requiring further validation in larger, multi-institution samples before widespread application.

## 1 Introduction

Since the post-pandemic era, China’s college graduate numbers have remained consistently high, with 11.58 million graduates in 2023 and 11.79 million in 2024 (Xie, 2023). The emergence of AI technologies like DeepSeek is further squeezing the survival space of traditional human labor, making ‘the most challenging job-hunting season in history’s reality.

Research on employment pressure and mental health among college students carries significant social implications and exploratory value for optimizing university admissions and ensuring stable employment outcomes (Xu and Zhong, 2002). As society becomes increasingly intelligent and employment prospects grow more complex, students face substantial psychological stress throughout their career development journey (Chen, 2019). This pressure not only affects their mental well-being but may also profoundly impact their professional growth. Chronic high-pressure states can trigger deep-seated anxiety and varying degrees of depression, even leading to self-denial and avoidance behaviors that severely compromise their social adaptability and quality of life (Mao, 2005). Conversely, poor mental health negatively impacts employability—students with psychological challenges often underperform in job searches (Sun et al., 2018), which further intensifies employment pressure and creates a vicious cycle (Tang et al., 2018). Therefore, in-depth research on the relationship between college students ‘employment pressure and mental health not only helps reveal the interaction mechanism between the two (Pan and Li, 2023), but also provides scientific evidence for families of current college students, regular universities, and a changing society. This aids in formulating effective intervention measures to alleviate students’ psychological stress and enhance their employability. Such efforts are of great significance for the personal growth of college students, social harmony, and sustainable development (Ministry of Human Resources and Social Security et al., 2024).

## 2 Purpose of research

The College Student Employment Stress Survey serves as a vital tool for assessing current employment conditions and psychological well-being among university students. This survey systematically collects data on stress sources, psychological experiences, career planning, and coping strategies encountered during job search processes. Furthermore, the findings provide scientific evidence for universities to optimize career guidance services, adjust curricula, and enhance mental health education. By conducting regular employment stress surveys, institutions can dynamically track evolving trends in student employment pressures, promptly identify issues, and implement targeted measures. This approach effectively alleviates employment-related stress, thereby promoting both mental health and career development among students.

This article aims to measure anxiety factors and explore the effectiveness of a self-made questionnaire based on the level of employment pressure among college students. The goal is to understand the causes, depth, and breadth of current employment pressure among college students, and to provide effective career planning basis for college employment guidance departments through reasonable exploratory research.

## 3 Development of the College Students’ Employment Stress Questionnaire

### 3.1 Research methods

Based on the open-ended questionnaire survey results, interviews, and literature review, we developed the initial draft of the College Student Employment Stress Questionnaire. Subsequently, a randomized sampling method was employed to conduct a blind test with 130 undergraduates from regular universities. Through exploratory factor analysis, we established the preliminary questionnaire structure. Finally, a controlled test was administered to 250 students, followed by confirmatory factor analysis to validate the questionnaire’s structural integrity.

### 3.2 Questionnaire design

#### 3.2.1 Subject

Open-ended Questionnaire: 30 students from Zhejiang University of Science and Technology (23 males, 7 females).

Interview method: 15 students from Zhejiang University of Science and Technology (10 male, 5 female).

Questionnaire: 130 questionnaires were sent out, 113 valid questionnaires were collected (88 male, 25 female, 22 urban, 91 rural, 25 party members, 88 non-party members, 10 confirmed workers, 103 unconfirmed workers).

Confirmatory factor analysis: 250 questionnaires were distributed, 234 valid questionnaires were collected (169 male, 65 female, 64 urban, 170 rural, 54 party members, 180 non-party members, 20 confirmed workers, 214 unconfirmed workers).

#### 3.2.2 Research methodology

Open-ended Questionnaire—Used to identify key issues regarding employment stress among college students.

College Students’ Employment Pressure Questionnaire: Based on the results of open questionnaires, it is used to evaluate the employment pressure of students.

### 3.3 Research procedures

#### 3.3.1 Questionnaire development

Through open questionnaires and interviews, the employment pressure of college students was integrated. Based on the existing research, the questionnaire was designed with 6 aspects and 31 questions. The 5-point scoring method (0 = no pressure, 4 = very high pressure) was adopted, and the higher the score, the greater the pressure. The results are shown in Table 1.

**Table 1:** College students’ employment stress questionnaire.

Number	Title
1	Poor appearance (e.g., body shape, facial features)
2	The family cannot provide strong work support
3	Financial crisis, professional social demand decreases
4	Parents have high expectations for their own employment
5	Their performance in their major subjects are not good.
6	relatives of no social standing in the family
7	There are many highly educated job seekers in the society, and the pressure is great
8	The career planning courses offered by the Institutional Resources are not well-designed.
9	Students have high expectations of themselves
10	The academic performance in university is not satisfactory.
11	The family’s financial situation is average.
12	Large number of graduates, fierce competition
13	The academic atmosphere in the Institutional Resources is not very strong.
14	Bachelor degree, lack of competitiveness in job search
15	I interviewed with several companies, but received no further response.
16	The family lacks a favorable social background.
17	Few jobs are available for the major studied
18	The unreasonable arrangement of professional courses in colleges and the low quality of teaching in colleges and universities
19	They hope to stay in a big city or an economically developed Regional Factors.
20	Their gender (e.g., female) does not match the job requirements for this field
21	Several organizations have tried it, but none have officially hired after the trial period.
22	Worried about being separated from your lover after work
23	There is not much social demand for this major
24	Institutional Resources do not give much attention to the cultivation of students’ hobbies
25	Poor computer or English skills
26	There are few corresponding social jobs for this major
27	The Institutional Resources does not provide much employment information or support.
28	Friends have high expectations for securing better jobs.
29	Personality introversion (or other introversion) is not conducive to job-hunting
30	The university I attended was not a prestigious institution and lacked recognition in society.
31	No internship experience, lacking relevant professional work experience

Following item analysis and exploratory factor analysis, the initial 31-item pool was reduced and reorganized. A total of 23 items were retained for the formal questionnaire. Table 2 presents a crosswalk between the initial items (V1–V31) and the final items (M1–M23), along with the rationale for deletion.

**Table 2:** Crosswalk of initial items (V1–V31) and final items (M1–M23).

Original item	Content summary	Retained	Final item	Assigned dimension	Reason for deletion
V1	Poor appearance (e.g., body shape, facial features)	Yes	M19	Personal Qualities	
V2	Family cannot provide strong work support	Yes	M1	Family Support	
V3	Financial crisis, professional social demand decreases	No			Cross-loaded
V4	Parents have high expectations for own employment	Yes	M4	Family Support	
V5	Performance in major subjects not good	Yes	M20	Personal Qualities	
V6	No relatives of social standing in family	Yes	M6	Institutional Resources	
V7	Many highly educated job seekers in society	Yes	M2	Job Market & Major Utility	
V8	Career planning courses offered by school are not well-designed	Yes	M23	Institutional Resources	
V9	Students have high expectations of themselves	No			Cross-loaded
V10	Academic performance in university not satisfactory	Yes	M8	Personal Qualities	
V11	Family’s financial situation is average	Yes	M7	Family Support	
V12	Large number of graduates, fierce competition	Yes	M5	Job Market & Major Utility	
V13	Academic atmosphere in school not very strong	Yes	M10	Institutional Resources	
V14	Bachelor degree, lack of competitiveness in job search	No			Factor loading < 0.45
V15	Interviewed with several companies, no further response	No			Factor loading < 0.45
V16	Family lacks favorable social background	Yes	M12	Family Support	
V17	Few jobs available for the major studied	Yes	M11	Job Market & Major Utility	
V18	Unreasonable arrangement of professional courses, low teaching quality	Yes	M14	Institutional Resources	
V19	Hope to stay in big city or economically developed area	Yes	M3	Regional Factors	
V20	Gender (e.g., female) does not match job requirements	No			Factor loading < 0.45
V21	Several organizations tried, none officially hired after trial period	No			Factor loading < 0.45
V22	Worried about being separated from lover after work	Yes	M22	Regional Factors	
V23	Not much social demand for this major	Yes	M13	Job Market & Major Utility	
V24	Schools do not give much attention to cultivation of hobbies	No			Factor loading < 0.45
V25	Poor computer or English skills	No			Factor loading < 0.45
V26	Few corresponding social jobs for this major	Yes	M16	Job Market & Major Utility	
V27	School does not provide much employment information or support	No			Cross-loaded
V28	Friends have high expectations for securing better jobs	No			Low communality
V29	Personality introversion not conducive to job-hunting	Yes	M21	Personal Qualities	
V30	University attended was not prestigious, lacked recognition	No			Cross-loaded
V31	No internship experience, lacking relevant work experience	Yes	M17	Personal Qualities	

#### 3.3.2 College Student Employment Pressure Survey’

The study administered questionnaires to senior and junior students (employment pressure-sensitive group), distributing 130 copies and receiving 113 valid responses. SPSS 28.0 was used for initial item analysis followed by exploratory factor analysis. For confirmatory factor analysis, 250 questionnaires were distributed, with 234 valid responses collected for factor analysis using AMOS 21.0.

### 3.4 Research results

#### 3.4.1 Analysis of the questionnaire items

SPSS 28.0 was used to analyze the data, calculate the total score of the scale and the average score of each item, and perform item analysis. Items with a correlation coefficient less than 0.30 with the total score were removed. After deletion, the results showed that the correlation coefficient of all items was greater than 0.30, so no items needed to be deleted (Table 3).

**Table 3:** Item analysis of college students’ employment pressure questionnaire.

Question number	Correlation with the total score of the scale
V1	0.459^**^
V2	0.674^**^
V3	0.711^**^
V4	0.587^**^
V5	0.571^**^
V6	0.745^**^
V7	0.546^**^
V8	0.613^**^
V9	0.645^**^
V10	0.649^**^
V11	0.736^**^
V12	0.732^**^
V13	0.687^**^
V14	0.693^**^
V15	0.661^**^
V16	0.726^**^
V17	0.659^**^
V18	0.591^**^
V19	0.515^**^
V20	0.493^**^
V21	0.534^**^
V22	0.501^**^
V23	0.702^**^
V24	0.587^**^
V25	0.521^**^
V26	0.727^**^
V27	0.639^**^
V28	0.623^**^
V29	0.672^**^
V30	0.665^**^
V31	0.543^**^

** indicates *p* < 0.01, meaning the result is statistically significant at the 0.01 level.

#### 3.4.2 Exploratory factor analysis of the prediction questionnaire

The factor analysis was initially conducted using principal component analysis and the maximum variance method from the orthogonal rotation method, with the results presented in Table 4. Principal Component Analysis (PCA) was selected for this exploratory phase given the practical objective of data reduction and the maximization of explained variance in the initial item pool. While Principal Axis Factoring is often recommended for identifying latent constructs, PCA was deemed appropriate for the preliminary development of a concise measurement instrument. Nonetheless, this choice represents a methodological limitation, and future confirmatory studies should employ factor analytic methods specifically designed for latent variable modeling. After evaluating both the results and the inherent significance of the original items, five factors were identified, accounting for a cumulative total variance of 64.46%. The first factor comprises items 2, 4, 6, 11, and 16; the second factor includes items 17, 24, 25, 26, and 27; the third factor covers items 1, 5, 10, and 29; the fourth factor involves items 19,22, and 23; and the fifth factor includes items 8, 13, and 18. (The item numbers mentioned above correspond to those in Table 1).

**Table 4:** Exploratory factor analysis of the college students’ employment stress questionnaire.

Project	F1	F2	F3	F4	F5
V3	0.744				
V7	0.742				
V4	0.709				
V14	0.658				
V12	0.638				
V30	0.636				
V16	0.632				
V13	0.584				
V19	0.578				
V2	0.576				
V6	0.576				
V11	0.552				
V20		0.728			
V22		0.715			
V9		0.618			
V21		0.606			
V29		0.522			
V15		0.420			
V18			0.735		
V8			0.594		
V25			0.529		
V27			0.481		
V17				0.691	
V26				0.674	
V31				0.570	
V24				0.567	
V23				0.561	
V28				0.474	
V1					0.733
V5					0.630
V10					0.617
Eigenvalue	19.521	11.035	10.624	10.398	8.882
Variance contribution rate	19.521	30.557	41.180	51.578	60.460

Table 4 presents the rotated component matrix from the principal component analysis. For clarity of presentation, only factor loadings greater than 0.45 are displayed. Items with loadings below this threshold or with substantial cross-loadings (loadings ≥0.30 on more than one factor) were considered for deletion and are not included in the final scale. Based on the analysis results and project significance evaluation, five factors were identified, collectively explaining 64.46% of the total variance. The specific factor distribution is as follows:

- Factor 1: Family Support (5 items, M1, M7, M12, M15, M4). This factor reflects the availability of financial resources, social connections, and parental expectations within the family context, all of which have been identified as significant determinants of employment stress in prior research.
- Factor 2: Job Market & Major Utility (5 items, M2, M5, M13, M16, M11). This factor captures the perceived supply–demand dynamics in the labor market, including the competitiveness of the graduate pool and the perceived market value of the student’s specific academic major.
- Factor 3: Personal Qualities (5 items, M21, M19, M8, M17, M20). This factor encompasses self-assessed competencies, academic performance, appearance, and personality traits that influence students’ confidence in their employability and job search prospects.
- Factor 4: Regional Factors (3 items, M3, M22, M18). This factor reflects preferences for and pressures related to geographic location, including the desire to remain in economically developed urban areas and concerns about separation from significant others due to job placement.
- Factor 5: Institutional Resources (4 items: M23, M6, M10, M14). This factor pertains to the perceived quality of university-provided career services, curriculum design, academic atmosphere, and institutional reputation in supporting students’ transition to the workforce.

(Item numbers refer to those in Table 1).

The formal questionnaire is attached as Appendix 1, and the reliability analysis of the formal questionnaire is shown in Table 5. After the strict analysis of the factors, the five factors are basically consistent with the scale structure, as shown in Table 6.

**Table 5:** Reliability analysis of the formal questionnaire.

Project	The reliability coefficients for each item on the scale
M1	0.929
M2	0.928
M3	0.929
M4	0.927
M5	0.928
M6	0.927
M7	0.928
M8	0.928
M9	0.927
M10	0.927
M11	0.928
M12	0.927
M13	0.928
M14	0.927
M15	0.926
M16	0.926
M17	0.927
M18	0.928
M19	0.928
M20	0.929
M21	0.930
M22	0.930
M23	0.927

**Table 6:** Results of KMO and Bartlett’s test for sphericity of formal questionnaires.

Indicators		
Kaiser–Meyer–Olkin Measurement of sampling appropriateness		0.868
Bartlett’s test of sphericity	Approximate chi-square distribution	2.326E3
	Variance	703
	Significance	0.000

It should be noted that the sample size for the exploratory factor analysis (*n* = 113) was relatively small relative to the initial item pool of 31 items (subject-to-item ratio approximately 3.6:1). While this ratio meets minimal requirements for exploratory work, it may affect the stability of the factor solution. Consequently, the results of the subsequent confirmatory factor analysis are critical for establishing the robustness of the scale structure, and replication with larger samples is recommended.

Finally, five factors were extracted, which explained 64.46% of the total variance, as follows:

Factor 1: Family (5 items: M1, M7, M12, M15, M4), mainly reflecting the Family Support conditions for college students’ employment pressure.

Factor Two: Job Market & Major Utility (5 items: M2, M5, M13, M16, M11), which reflects the direct impact of Job Market & Major Utility for talent on employment pressure for college graduates.

Factor 3: Personal Competence (4 items: M21, M19, M8, M17, M20) – The employment competitiveness of college students is critically influenced by their overall capabilities.

Factor 4: Geographic region (3 items: M3, M22, M18), which reflects how the local environment influences college students’ career choices and job Job Market & Major Utilities.

Factor 5: Institutional Resources (3 items: M23, M6, M10, M14), highlighting the Institutional Resources’ crucial role in developing employability and adapting to social roles.

#### 3.4.3 Reliability test of the questionnaire

As shown in Table 7, the overall alpha coefficient of the questionnaire is 0.955, with individual subscale alpha coefficients ranging from 0.493 to 0.842. It is important to note, however, that the “Institutional Resources” subscale (subsequently referred to as “Institutional Resources”) yielded a Cronbach’s *α* coefficient of only 0.493. This value falls below the commonly accepted threshold of 0.60 for minimally adequate internal consistency and substantially below the 0.70 standard for acceptable reliability. Consequently, this subscale does not demonstrate acceptable psychometric properties in its current form, and interpretation of scores on this dimension should be undertaken with considerable caution. Table 5 indicates that the reliability of individual items shows minimal variation compared to the overall reliability, demonstrating good reliability of the Full Scale. Table 8 further reveals that the correlation coefficients between subscales range from 0.550 to 0.737, while the subscale-to-total-reliability correlation coefficient ranges from 0.797 to 0.898, further confirming the scale’s high reliability.

**Table 7:** Reliability statistics of the total form and subscales of the formal questionnaire.

Dimension	Full Scale	Personal Quality	Family	Job Market & Major Utility	Regional Factors	Institutional Resources
Coefficient A	0.955	0.842	0.773	0.727	0.735	0.493

**Table 8:** The correlation between the subscales of the formal questionnaire.

Dimension	Personal Quality	Family	Job Market & Major Utility	Regional Factors	Institutional Resources	Full Scale
Personal Quality	1					
Family	0.665^**^	1				
Job Market & Major Utility	0.550^**^	0.622^**^	1			
Regional Factors	0.589^**^	0.737^**^	0.619^**^	1		
Institutional Resources	0.564^**^	0.701^**^	0.580^**^	0.631^**^	1	
Full Scale	0.842^**^	0.898^**^	0.797^**^	0.832^**^	0.803^**^	1

** indicates *p* < 0.01, meaning the result is statistically significant at the 0.01 level.

#### 3.4.4 Validity test of the questionnaire

##### 3.4.4.1 Content validity

The validity of test content refers to whether the test content can fully represent the scope of the test and reflect the psychological traits of the target, which depends on the clarity of the content scope and the representativeness of the sample. On the basis of obtaining sample data of domestic students through interviews and surveys, and referring to existing research results, a preliminary test form was developed. After further analysis and verification, a questionnaire was formed, indicating that the questionnaire has good content validity.

##### 3.4.4.2 Confirmatory factor analysis

To verify the structural validity of the five-factor model derived from the exploratory factor analysis, a confirmatory factor analysis (CFA) was conducted using AMOS 21.0 with Maximum Likelihood estimation. The model specified 23 observed items loading onto their respective five latent factors (Personal Qualities, Family Support, Job Market & Major Utility, Institutional Resources, and Regional Factors), with the latent factors allowed to correlate freely. The analysis was performed on the formal testing sample (*n* = 234).

**Figure 1:**
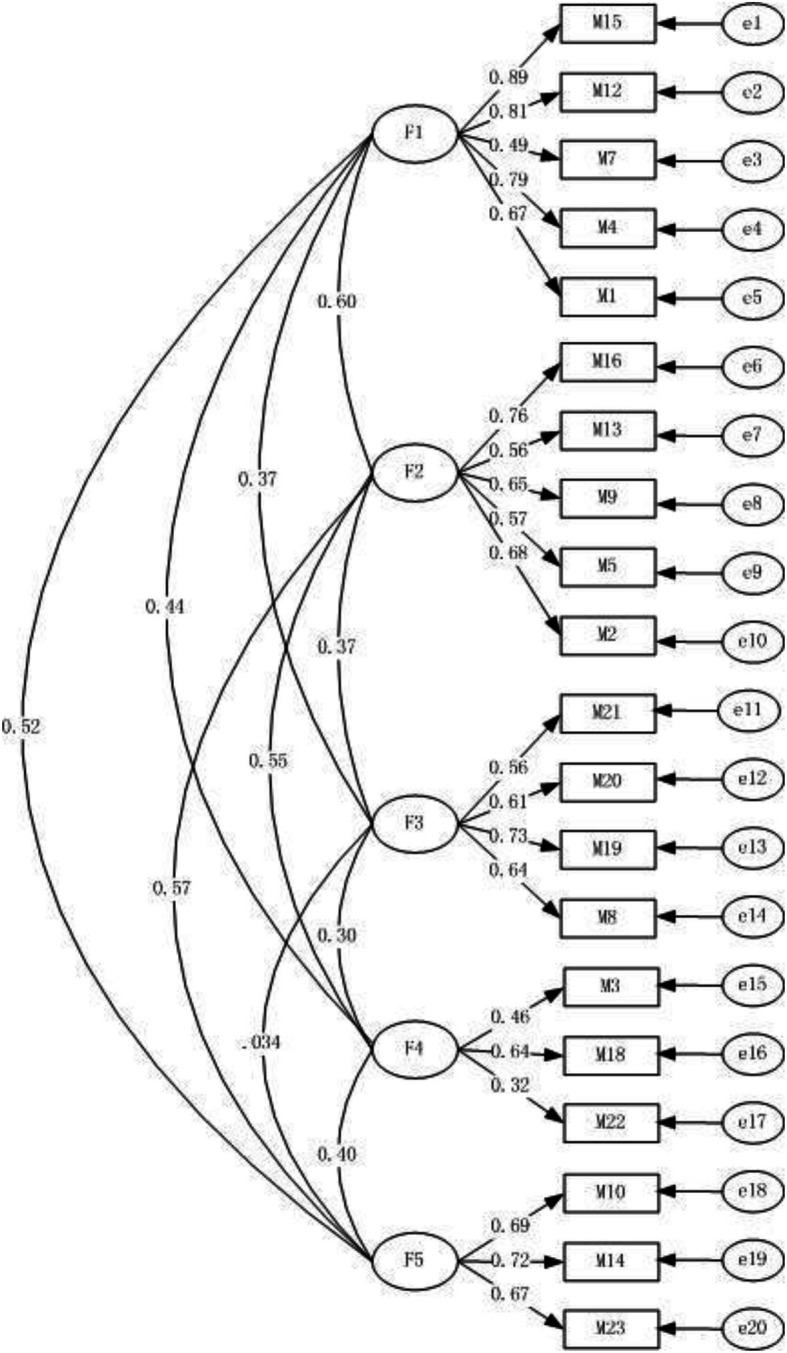
Results of confirmatory factor analysis.

The CFA model is presented in Figure 1, and the standardized parameter estimates are displayed.

As shown in Table 9, the model fit indices were not satisfactory. The *χ*^2^/df ratio was 3.942, exceeding the liberal threshold of 3.0. The RMSEA value was 0.113, which is above the conventional 0.08 cutoff for acceptable fit. Furthermore, the incremental fit indices (CFI = 0.79, TLI = 0.74) fell substantially short of the recommended 0.90 standard. These results indicate that the hypothesized five-factor model received only marginal support in the current sample. The structural validity of the scale therefore requires further investigation and refinement, and the conclusion that the model demonstrates good fit cannot be sustained. Possible reasons for the marginal fit include the modest sample size, the low internal consistency of the Institutional Resources subscale, and potential item-level misspecifications. Future research should consider model modification, item revision, or cross-validation with larger and more diverse samples before the scale can be confidently recommended for applied use.

**Table 9:** Model fitting index.

*χ* ^2^	df	*χ*^2^/df	TLI	IFI	RMSEA	NFI	CFI
630.74	160	3.942	0.74	0.794	0.113	0.742	0.79

##### 3.4.4.3 Validity and reliability

Literature research shows that college graduates are prone to psychological problems such as anxiety, dependence, and inferiority complex in the fiercely competitive job market. Ouyang et al. (2004) pointed out that employment pressure often leads to feelings of inferiority, conflict, and anxiety among college students, which may harm their mental health. The study also found that moderate employment pressure can directly trigger psychological anxiety, but does not affect normal behavioral functions (Feng and Zhou, 2002). Liu and Zhang (2022) and Yang (2021) pointed out that employment pressure is the main source of psychological and social pressure for college graduates; Fan et al. (2022) emphasized that the anxiety caused by employment pressure deserves attention.

Accordingly, the anxiety status of participants in the present study was assessed using the Self-Rating Anxiety Scale (SAS), a well-established instrument suitable for adults experiencing anxiety symptoms. The SAS is widely used in both clinical and research settings due to its ease of administration and robust psychometric properties.

The analysis, as shown in Table 10, revealed a positive but weak correlation between the Employment Stress Scale total score and the SAS (*r* = 0.151, *p* < 0.05). This finding provides preliminary evidence of criterion-related validity, indicating that students reporting higher employment stress also tend to report slightly elevated anxiety symptoms. However, the small magnitude of the correlation suggests that employment stress and general anxiety are distinct constructs with only modest overlap in this non-clinical sample. This is theoretically plausible, as employment stress during the job search period may be a normative developmental challenge rather than a pathological state. Nevertheless, the weak association underscores the need for additional validation efforts using a broader array of criterion measures in future research.

**Table 10:** The correlation matrix of college students’ employment pressure, self-concept and anxiety.

Employment stress	Employment pressure	Concept of the self	Anxious
Employment pressure	1		
Concept of the self	0.068	1	
Anxious	0.151^*^	0.253^**^	1

** indicates *p* < 0.01, meaning the result is statistically significant at the 0.01 level. “*”indicates *p* < 0.05, meaning the result is statistically significant at the 0.05 level.

## 4 Discussion

The “College Student Employment Stress Scale” developed in this study was based on a sample of 234 undergraduate students from Zhejiang University of Science and Technology (ZUST) and resulted in a 23-item formal questionnaire comprising five dimensions: Personal Qualities, Family Support, Job Market & Major Utility, Institutional Resources, and Regional Factors. It is essential to emphasize that the scale was validated exclusively within a single institution in Zhejiang Province, China. Consequently, the findings should not be generalized to all Chinese college students or to international student populations without further cross-validation in diverse institutional and regional contexts. Despite this limitation, the scale provides a multidimensional framework for understanding the sources of employment stress among university students. The cumulative variance explained by the five factors was 64.46%, indicating that the scale captures a substantial portion of the variance in self-reported employment stress. By incorporating personal, familial, market-based, institutional, and Regional Factors, the instrument offers a more comprehensive assessment than unidimensional measures of job search anxiety.

Research has shown that the employment pressure of college students varies significantly depending on their grade level. Due to difficulties in employment internships and high expectations, senior students experience significantly higher levels of pressure compared to lower and middle grade students (Ma, 2017; Wang, 2008). Yang (2018) research also points out that lower grade students also face pressure due to high curriculum pressure and high employment expectations. Therefore, this study covers students of all grades, and this scale serves as a basic measurement tool, laying the foundation for further enhancing the depth and breadth of research in the future. Based on the five dimensions and 32 questions of the scale, universities can carry out more effective employment guidance work, including strengthening theoretical courses and practical skills training, and enhancing students’ comprehensive competitiveness; Combining mental health education with employment guidance to enhance students’ ability to cope with psychological stress; At the societal level, it is necessary to strengthen cooperation between Institutional Resources and enterprises, provide internship positions and guidance from industry experts, and improve employment policies; In terms of family, parents should be encouraged to communicate with their children and alleviate student stress through care and concern; Students themselves need to have a correct attitude, deeply understand the industry rules in society, and be fully prepared for employment.

This study has several important limitations. First, the sample was restricted to a single institution, and the sample size for the exploratory factor analysis was modest, which may affect the stability of the factor solution. Second, the confirmatory factor analysis yielded marginal to poor fit indices, indicating that the hypothesized five-factor model was not adequately supported by the data. Third, the “Institutional Resources” subscale demonstrated unacceptably low internal consistency (*α* = 0.493). This poor reliability likely reflects the conceptual heterogeneity of the items within this dimension. Future revisions should consider refining these items or removing the subscale entirely. Fourth, criterion validity evidence was limited to a single, weak correlation with the Self-Rating Anxiety Scale. Future research should expand the sample and incorporate additional validation measures.

## Data Availability

The original contributions presented in the study are included in the article/supplementary material, further inquiries can be directed to the corresponding author.
